# Activation of the MAC1-ERK1/2-NOX2 Pathway Is Required for LPS-Induced Sustaining Reactive Microgliosis, Chronic Neuroinflammation and Neurodegeneration

**DOI:** 10.3390/antiox11061202

**Published:** 2022-06-20

**Authors:** Shih-Heng Chen, Shuangyu Han, Chih-Fen Hu, Ran Zhou, Yun Gao, Dezhen Tu, Huiming Gao, Jing Feng, Yubao Wang, Ru-Band Lu, Jau-Shyong Hong

**Affiliations:** 1 Neurobiology Laboratory, National Institute of Environmental Health Sciences, National Institutes of Health, Research Triangle Park, NC 27709, USA; hsyu1297@tmu.edu.cn (S.H.); caperhu@mail.ndmctsgh.edu.tw (C.-F.H.); ranniezhou4@163.com (R.Z.); gaoyun@nicemice.cn (Y.G.); tudezhen@sibcb.ac.cn (D.T.); yubaowang2020@hotmail.com (Y.W.); hong3@niehs.nih.gov (J.-S.H.); 2 Respiratory Department, Tianjin Medical University General Hospital, Tianjin 300052, China; zyyhxkfj@126.com; 3 Department of Pediatrics, Tri-Service General Hospital, National Defense Medical Center, Taipei 10086, Taiwan; 4 MOE Key Laboratory of Model Animal for Disease Study, Model Animal Research Center, Institute for Brain Sciences, Nanjing University, Nanjing 210061, China; gaohm@nicemice.cn; 5 Institute of Infectious Diseases, The Second Hospital of Tianjin Medical University, Tianjin 300211, China; 6 Institute of Behavioral Medicine, National Cheng Kung University, Tainan 70101, Taiwan; rblu@mail.ncku.edu.tw

**Keywords:** MAC1 receptor, neuroinflammation, oxidative stress, ERK1/2, NADPH oxidase, TLR4

## Abstract

Recent studies suggest that improper resolution of acute neuroinflammation may lead to long-lasting low-grade chronic neuroinflammation and drive progressive neurodegeneration. However, the molecular mechanism underlying the transition from acute to chronic neuroinflammation remains unclear. The main purpose of this study was to search for potential pathways mediating LPS-elicited chronic neuroinflammation and resultant neurodegeneration. Using microglia cultures prepared from C57BL/6J, MAC1-deficient, and MyD88-deficient mice, the initial study showed that activation of TLR-4 is not sufficient for maintaining chronic neuroinflammation despite its essential role in LPS-initiated acute neuroinflammation. Opposite to TLR-4, our studies showed significantly reduced intensity of chronic neuroinflammation, oxidative stress, and progressive loss of nigral dopaminergic neurons in MAC1-deficient neuron/glial cultures or mice stimulated with LPS. Mechanistic studies revealed the essential role ERK1/2 activation in chronic neuroinflammation-elicited neurodegeneration, which was demonstrated by using an ERK1/2 inhibitor in neuron-glial cultures. Taken together, we propose a key role of the MAC1-NOX2-ERK1/2 signaling pathway in the initiation and maintenance of low-grade chronic neuroinflammation. Continuing ERK1/2 phosphorylation and NOX2 activation form a vicious feedforward cycle in microglia to maintain the low-grade neuroinflammation and drive neurodegeneration.

## 1. Introduction

Neuroinflammation is a self-defense mechanism to combat pathogen infections or repair the injuries in the central nervous system (CNS). This reaction is initially mediated by microglia and shaped by reactive astroglia and other infiltrating leukocytes [1,2]. Acute neuroinflammation is typically initiated by detecting the pathogen-associated molecular patterns (PAMPs) from microorganisms or the damage-associated molecular patterns (DAMPs) molecules released from injured or dying cells in the brain. Once acute neuroinflammation is launched, microglia are recruited to clear these signals by preventing infection or restoring injuries in the brain [3]. Microglial Toll-like receptors (TLRs) are the most extensively studied signaling pathway mediating the pathological effects of both PAMPs and DAMPs [4]. PAMPs derived from Gram-negative bacteria or its endotoxin lipopolysaccharide (LPS) act on TLR4 [5]. Various DAMPs are known to act on different TLRs: α-synuclein can act on TLR2 [6], and both β-amyloid [7,8,9] and HMGB-1 [10,11] can act on TLR2, TLR4, and TLR9. Activation of TLRs signals various adaptor molecules to stimulate the production of proinflammatory cytokines and chemokines. Acute neuroinflammatory responses are typically transient and help restore CNS homeostasis. However, in pathological conditions, neuroinflammation may continue and become long-lasting chronic neuroinflammation [2]. The mechanism by which transition from acute to chronic neuroinflammation in disease states remains largely unknown.

Recent evidence indicates that chronic low-grade neuroinflammation plays a crucial role in the pathogenesis of neurodegenerative diseases, such as Alzheimer’s disease and Parkinson’s disease [12]. However, the molecular mechanism mediating chronic neuroinflammation is less clear. Studies showed that brains from patients with neurodegenerative diseases or that have undergone normal aging had greater expression of TLR and proinflammatory genes likely due to increased DAMPs during neurodegeneration [13,14]. These results led to the notion that an aberrant TLR activation contributes to the process of aging and neurodegenerative diseases. However, it is not clear whether the initial activation of microglial TLRs, which is critical for the initiation of acute inflammation in the brain, is sufficient for the maintenance of chronic neuroinflammation. Based on published literature and our previous studies, we studied the possibility that MAC1 (also known as CD11b/CD18, complement receptor 3 (CR3), or αMβ2) could be a closely linked receptor in LPS-induced chronic neuroinflammation. Compared with TLR-4, much less is known regarding the role of MAC1 in mediating reactive microgliosis during sustained chronic neuroinflammation. We have previously reported that the LPS exerts less neurotoxicity in primary mouse midbrain neuron-glial cultures prepared from MAC1-deficient mice than that from wild-type mice, suggesting a role of MAC1 in LPS-elicited chronic reactive microgliosis and in driving inflammation-mediated neurodegeneration [15]. This study aimed to further elucidate the distinct roles of MAC1 and TLR receptors in mediating LPS induced acute vs. chronic neuroinflammation and subsequent neurodegeneration using both in vivo and in vitro studies. This study showed that acute activation of TLR receptors alone is not sufficient; instead, activation of MAC1 is necessary for sustaining chronic neuroinflammation and leading to neurodegeneration. We also uncovered a critical signaling pathway, including NADPH oxidase 2 (NOX2)/superoxide/extracellular signal-regulated kinase (ERK)1/2 in mediating the effects of MAC1 in maintaining self-propelling reactive microgliosis found in chronic neuroinflammation.

## 2. Materials and Methods

### 2.1. Animals

C57BL/6J, B6.129S4-Itgam<tm1Myd>/J (MAC1 deficient), B6.129P2(SJL)-Myd88<tm1.1Defr>/J (MyD88 deficient), and B6.129S-Cybb<tm1Din>/J (gp91 deficient) mice were generated by our institute’s animal husbandry staff using breeders obtained from Jackson Laboratories (Bar Harbor, ME, USA). Mouse dams were housed in polycarbonate cages in animal facilities with controlled environmental conditions with a 12 h artificial light-dark cycle and provided fresh deionized water and NIH 31 chow ad libitum. All animal procedures were approved by the Institutional Animal Care and Use Committee (Animal Study Protocol # 86-21) and conducted in strict accordance with the National Institutes of Health animal care and used guidelines. A single systemic injection of lipopolysaccharide (LPS) (15 × 106 EU/kg, i.p., *Escherichia coli* 0111: B4, Sigma-Aldrich, St. Louis, MO, USA) was administered to 8–12 weeks old C57BL/6J and MAC1 KO mice (B6.C3-Tg [B6.129S4-Itgam<tm1Myd>/J]) mice. Mice used as vehicle control were injected with saline (5 mL/kg, i.p.). Mice were sacrificed at different time points via cervical dislocation, and the brain tissues were collected for further analysis.

### 2.2. Reagents

GBR12935 and urea-hydrogen peroxide tablets were purchased from Sigma-Aldrich (St. Louis, MO, USA). Lipopolysaccharide (LPS; *E. coli* strain O111: B4) was purchased from Calbiochem (San Diego, CA, USA). Cell culture ingredients were obtained from Life Technologies (Grand Island, NY, USA). U0126 was purchased from Cell Signaling Technology (Danvers, MA, USA). Antityrosine hydroxylase (TH) was purchased from Chemicon (Billerica, MA, USA), and antibody diluent was purchased from DAKO (Carpinteria, CA, USA). Anti-p47*^phox^* antibody was purchased from Millipore (Temecula, CA, USA). Anti-gp91*^phox^* antibody was purchased from BD Biosciences (San Jose, CA, USA). Anti-GAPDH and anti-3-NT antibodies were purchased from Abcam (Cambridge, MA, USA). Alexa Fluor 488 and Alexa Fluor 594 goat antimouse IgG and peroxidase-conjugated antirabbit and antimouse secondary antibodies were purchased from Invitrogen (Carlsbad, CA, USA). Goat antirabbit biotinylated secondary antibody was purchased from Vector Laboratory (Burlingame, CA, USA).

### 2.3. Mesencephalic Neuron-Glia Culture

Rat mesencephalic neuron-glia cultures were prepared following protocols described previously [16,17]. Briefly, midbrain tissues were dissected from day 14 embryos and then gently triturated into the single-cell suspension. Cells were then seeded (5 × 10^5^ cells/well) in poly-D-lysine (20 μg/mL) precoated 24-well plates. The cultures were incubated at 37 °C in 5% CO_2_ for three days and then replenished with 500 μL of fresh maintenance media. Cultures were treated seven days after seeding.

### 2.4. Primary Cortical Mixed Glial Culture

Primary cortical mixed glial cultures were prepared from mouse pup brains at postnatal day 1-3, as previously described [16,17]. Briefly, the cortices were isolated, the meninges and blood vessels removed, the tissue gently dissociated through trituration, and the single-cell suspension plated on either 24-well plates or 96-well plates was precoated in poly-D-lysine (20 μg/mL) at 1 × 10^5^ cells/well or 5 × 10^4^ cells/well, respectively. Cells were maintained in DMEM-F12 (1:1) media supplemented with 10% heat-inactivated fetal bovine serum (FBS), 2 mM L-glutamine, 1 mM sodium pyruvate, 100 μM nonessential amino acids, 50 U/mL penicillin, and 50 μg/mL streptomycin. Media were refreshed every 3 days until they were experimentally treated 7 days after seeding.

### 2.5. Microglia-Enriched Cultures

Microglia-enriched cultures were prepared from primary mixed glial cultures as previously described [16,17]. Briefly, mixed glial cultures were plated on 150 cm^3^ flasks pre-coated in poly-D-lysine (20 μg/mL) at 5 × 10^7^ cells/flask and were maintained in DMEM-F12 media changed every three days for two weeks. At two weeks, microglia were shaken off at 180 rpm for 40 min and replated on glass-bottom culture dishes (MatTek, Ashland, MA, USA) precoated in poly-D-lysine (20 μg/mL) at 1 × 10^6^ cells/well for immunofluorescence staining after LPS stimulation.

### 2.6. RNA Analysis

Total RNA was extracted from the midbrain region of the mouse brains with Qiagen RNeasy Minikit and reverse transcribed with an oligo dT primer. Real-time PCR amplification was performed using SYBR Green PCR Master Mix and an ABI 7900 HT Sequence Detection System (Applied Biosystems, Foster City, CA, USA) according to manufacturer’s protocols. The primers were designed using Vector NTI software (v.11, Invitrogen, Carlsbad, CA, USA) and validated for efficacy through melting curve analyses. Mouse GAPDH Forward (5′- TTCAACGGCACAGTCAAGGC-3′; 300 nM), Mouse GAPDH Reverse (5′- GACTCCACGACATACTCAGCACC-3′; 300 nM), Mouse TNF-α Forward (5′ GACCCTCACACTCAGATCATCTTCT 3′; 300 nM), Mouse TNF-α Reverse (5′ CCTCCACTTGGTGGTTTGCT 3′; 900 nM), Mouse GAPDH (GenBank: NM_008084), mouse TNF-α (GenBank: NM_013693.3). Amplifications were performed at 95 °C for 10 s, 55 °C for 30 s, and 72 °C for 30 s for 40 cycles. All samples were tested in triplicate from at least three independent experiments and normalized with GAPDH using the 2^−ΔΔCt^ method. Fold changes for each treatment were normalized in percentage to the maximum expression.

### 2.7. Dopamine Uptake Assay

The [^3^H] dopamine (DA) uptake assay was performed as described previously [18]. Briefly, the rate of uptake of radiolabeled DA by DAnergic neuron cultures was measured for 21 min at 37 °C. Cells were washed and lysed to release internalized radiolabeled DA and quantified with a liquid scintillation counter (Tri-Carb 4000; Packard, Meriden, CT, USA). Nonspecific [^3^H] DA uptake was accounted for by competitively inhibiting DA uptake with 20 μM of GBR12935.

### 2.8. Immunocytochemical and Immunofluorescence Staining

Immunostaining was performed as described previously. Mouse brains were cut into 35 μm sections on a horizontal sliding microtome. The free-floating brain slices were treated with 1% hydrogen peroxide for 10 min and incubated for 20 min with blocking solution (BSA 1%/Triton X-100 0.4%/Normal Goat Serum 4% in PBS). Brain slices were immunostained overnight at 4 °C with rabbit polyclonal antibody against tyrosine hydroxylase (TH; 1:5000) or ionized calcium binding adaptor molecule 1 (Iba-1) (1:4000) in antibody diluent. Brain slices were washed for 10 min in PBS (three times) and incubated for two hours with PBS containing 0.3% Triton X-100 and a biotinylated secondary antibody (goat antirabbit antibody, 1:227; Vector Laboratory, Burlingame, CA, USA). After washing (three times) with PBS, the brain slices were incubated for one hour with the Vectastain ABC reagents (Vector Laboratory, Burlingame, CA, USA) diluted in PBS containing 0.3% Triton X-100. To visualize the signal, the brain slices were incubated with 3,3′-diaminobenzidine and urea-hydrogen peroxide tablets dissolved in water. To monitor DA neurodegeneration, two individuals double-blind counted the number of TH-immunoreactive (TH-IR) neurons in the SN pars compacta (SNpc) of eight evenly spaced brain sections from a series of 24 sections that covered the entire SN. TH-positive cells were manually counted under a microscope (Nikon, model DIAPHOT, Garden City, NY, USA).

For immunofluorescence staining, microglia-enriched cultures were stained with anti-p47*^phox^* antibody, and brain slices were stained with anti-3-NT antibody. Microglia-enriched cultures were fixed with 3.7% formaldehyde in PBS for 20 min. Cultures and brain slices were incubated for 20 min in blocking solution (BSA 1%/Triton X-100 0.4%/Normal Goat Serum 4% in PBS) to block nonspecific binding. Cultures were immunostained overnight at 4 °C with polyclonal rabbit antibody against p47*^phox^* (1:1000) diluted in antibody diluent. The brain slices were immunostained overnight at 4 °C with mouse monoclonal antibody against 3-NT (1:200). The signals were detected and visualized using Alexa Fluor 488 goat antirabbit IgG (1:750) and Alexa Fluor 594 goat antimouse IgG (1:750) secondary antibodies for cultures and brain slices, respectively. The images were acquired using a multiphoton laser-scanning microscope Zeiss 710. The fluorescence intensity was quantified using ImageJ software (NIH, Bethesda, MD, USA).

### 2.9. Western Blot Analysis

The protein extracts from cultured cells were homogenized in radioimmunoprecipitation assay (RIPA) lysis buffer (50 mM Tris-HCl, pH 8.0, 150 mM NaCl, 5 mM EDTA, 1% NP-40, 0.5% sodium deoxycholate, 0.1% SDS, and 1:100 protease inhibitor cocktail). Protein concentrations were determined using the bicinchoninic acid assay (Pierce) and denatured in protein loading buffer. Equal amounts of protein were separated by 4% to 12% Bis-Tris Nu-PAGE gel and transferred to PVDF membranes (Bio-Rad, Hercules, CA, USA). Membranes were blocked with 5% nonfat milk and incubated with antibodies against p47*^phox^*, gp91*^phox^*, ERK1/2 (1:1000), or GAPDH (1:2500). Membranes were blocked with 5% BSA when incubated with antibodies against phosphor-ERK1/2 (1:2000). The protein bands were developed by incubating with horseradish peroxidase-conjugated secondary antibodies (Vector Laboratories, Burlingame, CA, USA) and an enhanced chemiluminescence substrate kit (Millipore, Billerica, MA, USA). The results were quantified by ImageJ software (NIH, Bethesda, MD, USA).

### 2.10. Statistics

Data are presented as the mean ± SEM. Comparisons between more than two groups were conducted using one-way ANOVA followed by Bonferroni’s post hoc multiple comparison test. Comparisons between more than two parameters were conducted using two-way ANOVA analysis followed by Bonferroni’s post hoc multiple comparison test. Data were analyzed using Prism (v6.00, GraphPad, San Diego, CA, USA). *p*-values less than or equal to 0.05 were considered statistically significant.

## 3. Results

### 3.1. MAC1-Deficiency Reduces LPS-Induced Chronic but Not Acute Brain Inflammation

**Figure 1:**
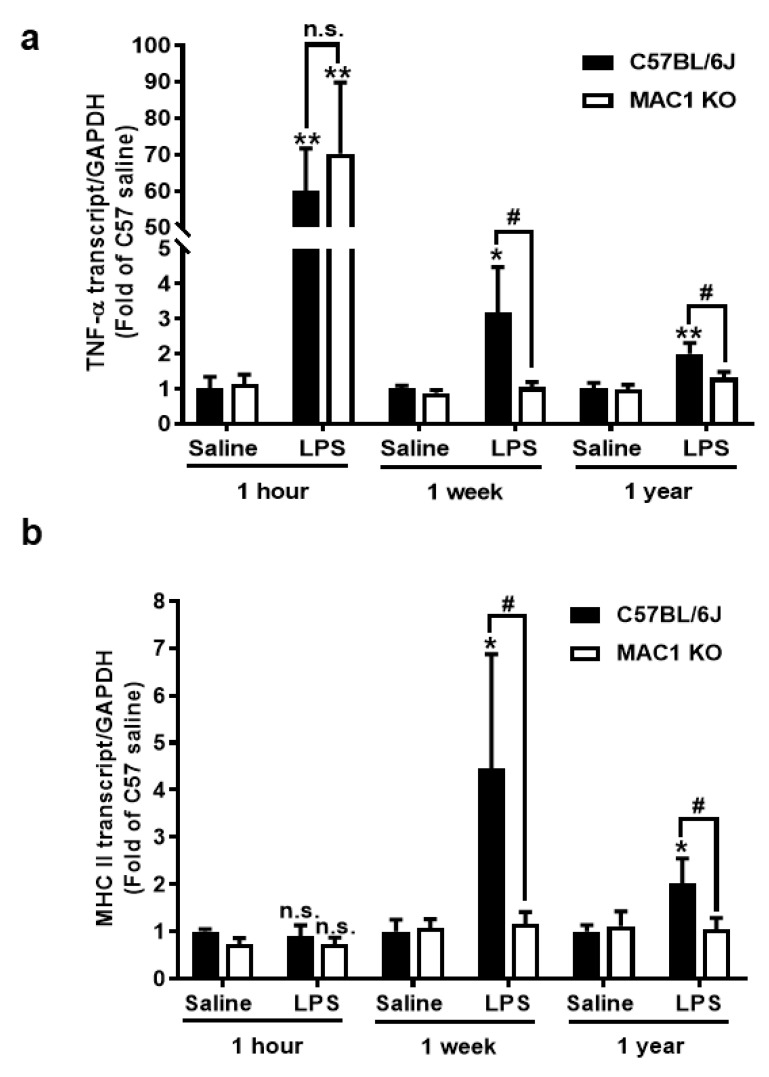

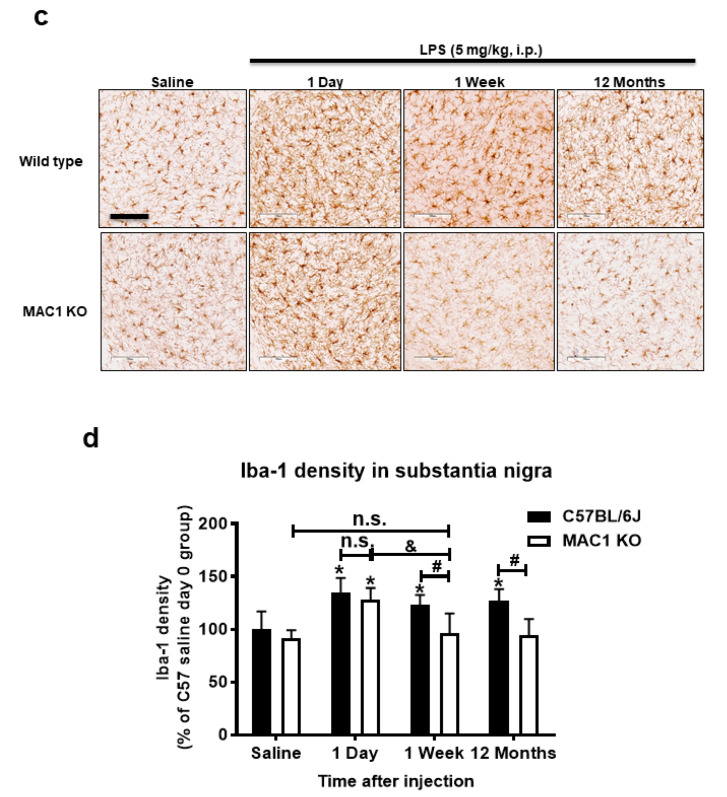
MAC1-deficiency reduces LPS-induced chronic but not acute brain inflammation. C57BL/6J and MAC1 KO mice were injected with saline or LPS (5 mg/kg, i.p) (N = 3 in each group and time point). The brain tissues were collected at 1 h, 7 days, and 12 months after injection for analysis of (**a**) TNF-α and (**b**) MHCII mRNA expression. (**c**) The brain slices from saline control or 1 day, 1 week, and 12 months after LPS injection were immunostained with microglia marker Iba-1, follow by (**d**) Iba-1 densitometry analysis of microglia in the substantia nigra. There are 3 brain slices stained in each group. * *p* < 0.05; ** *p* < 0.01; & *p* < 0.05; # *p* < 0.05.

To investigate the roles of the MAC1 receptor in the development and maintenance of chronic brain inflammation, we used our previously developed LPS mouse neurodegeneration model. Following a signal systemic injection of bacterial endotoxin LPS, mice developed acute neuroinflammation during the first few days. If inflammation is not resolved, it will transition to long-lasting chronic neuroinflammation, and delayed and progressive neurodegeneration occurs in different brain regions [19,20,21]. We compared the mRNA expression of an acute proinflammatory cytokine tumor necrosis factor alpha (TNF-α) gene and a late-expressed microglia activation marker MHC class II gene in wild-type and MAC1-deficient mouse brains at 1 h (for acute neuroinflammation), 7 days (for chronic neuroinflammation), and 12 months (for neuron loss studies) after LPS injection (5 mg/kg; ip). Similar increases in brain TNF-α mRNA were observed 1 h after LPS injection in both wild-type and MAC1-deficient mouse, suggesting a nonessential role of MAC1 in LPS-induced acute inflammation (Figure 1a). There was no increase of MHCII mRNA level at 1 h after LPS injection (Figure 1b). By contrast, the essential role of MAC1 became clear during the chronic inflammatory stages (1 week and 12 months points). mRNA levels of TNF-α and MHCII remained higher than the basal levels at these two-time points after LPS injection in wild-type mice. In contrast, mRNA for all these two genes returned to basal levels at one week and 12 months after LPS injection in MAC1-deficient mice.

Consistent with the gene expression results, morphological studies also showed a lack of sustained microglial activation in MAC1-deficient mice after LPS injection. One day after LPS injection, microglia are equally activated in both wild-type and MAC1 KO mouse brains as revealed by enhanced Iba-1 staining and a hypertrophied morphology. However, sustained microglial activation was observed 12 months after LPS injection only in wild-type but not in MAC1 KO mice. (Figure 1c,d). Taken together, these results support our hypothesis that LPS-initiated acute inflammation cannot be transitioned to chronic neuroinflammation without the presence of MAC1.

### 3.2. LPS-Elicited Loss of Dopaminergic Neurons Was Ameliorated in MAC1-Deficient Mouse Brains

**Figure 2:**
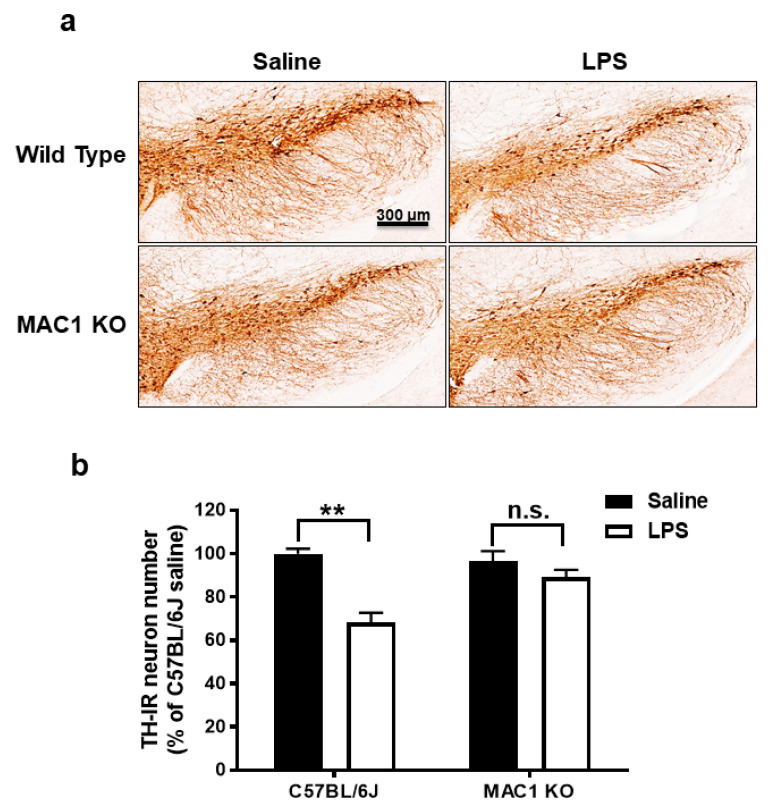
LPS-elicited loss of dopaminergic neurons was ameliorated in MAC1 KO mouse brains. (**a**) C57BL/6J and MAC1 KO mice were injected with saline or LPS for 12 months (WT control N = 3, WT LPS N = 5, MAC1 KO control N = 3, MAC1 KO LPS N = 5). Brains were removed for immunostaining with a DAergic neuron marker, tyrosine hydroxylase (TH). (N = 3) (**b**) Cell counting results. Scale bar = 300 μm; ** *p* < 0.01.

Our previous studies showed LPS-induced neuroinflammation-related progressive dopaminergic neurodegeneration in the substantia nigra starting from 7 months after injection [22]. To test whether MAC1 plays a key role in progressive neurodegeneration, we injected both wild-type and MAC1 KO mice with LPS (5 mg/kg, i.p.). Mice were euthanized at 12 months after LPS injection, and the number of tyrosine hydroxylase immune-positive neurons (TH-positive neurons) in the substantia nigra were counted. The results showed that the TH-positive neuron numbers in wild-type and MAC1 KO mice receiving vehicle injection are comparable. By contrast, a more significant loss of nigral TH-positive neurons was found in wild-type mice (35%) than that in MAC1 KO mice (less than 10%) (Figure 2).

### 3.3. Reactive Microgliosis Was Reduced in LPS-Treated MAC1- or NOX2-Deficient Neuron/Glia Cultures

**Figure 3:**
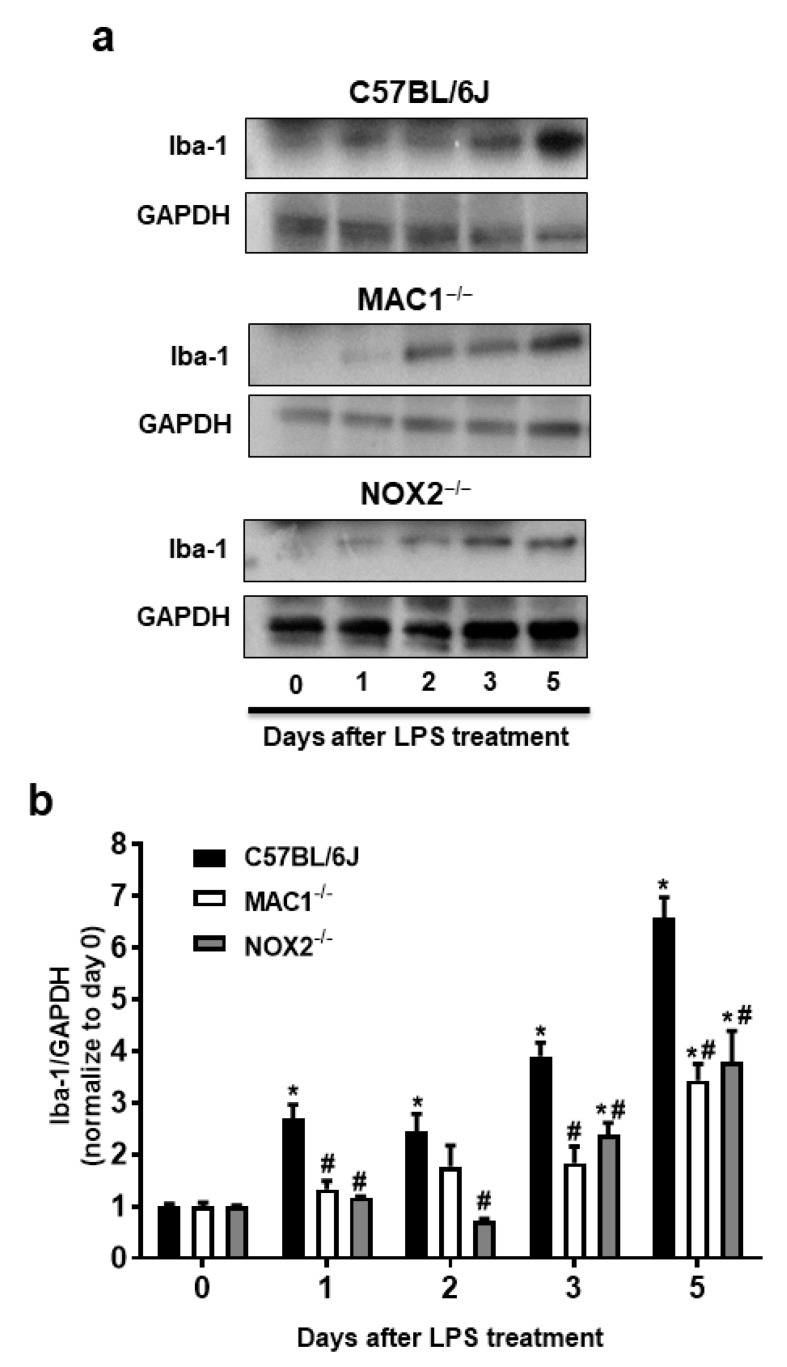
Reactive microgliosis was reduced in LPS-treated MAC1- or NOX-deficient neuron/glia cultures. (**a**) Neuron-glial cultures from wild type, MAC1 KO, and NOX2 KO mice were treated with LPS (20 ng/mL), and cell pellets were collected at various time points as indicated for Iba-1 detection. Quantification of Iba-1 in (**b**) wild type and MAC1 KO or wild type and NOX2 KO neuron-glial cultures. Data show mean ± SEM from 3 independent experiments. * *p* < 0.05; # *p* < 0.05.

Our previous studies have indicated that NOX2 is one of the important downstream singling molecules mediating actions of MAC1 during neuroinflammation by enhancing the production of superoxide free radicals [15,23,24]. Furthermore, studies also demonstrated that NOX2 contributes not only to the initiation but also the maintenance of persistent microglial activation [23]. NOX2-induced production of ROS and related oxidative stress are critical in chronic and progressive neurodegeneration [2,25]. The following studies aimed at elucidating the signaling pathways downstream of MAC1 receptor activation by using different types of midbrain primary neuron-glial cultures from mutant mice deficient in MAC1 and NOX2. A previous study demonstrated the interdependency of prolonged microglial activation (reactive microgliosis) and progressive neurodegeneration [2]. Moreover, damage-associated molecular patterns (DAMPs) released from damaged neurons, such as α-synuclein (α-syn) [26], β-amyloid (Aβ) [27] or high-mobility group box 1 (HMGB1) [23], could act on MAC1 to activate microglia. Therefore, we proposed that MAC1 is crucial in maintaining prolonged microglial activation. To test this hypothesis, we used neuron-glial mix cultures generated from wild-type and MAC1 KO mice. Western blot analysis showed a linear increase in the expression of microglial marker Iba-1 immunoreactivity from day 1 to day 5 after LPS treatment in neuron-glial cultures prepared from C57BL/6J (wild-type) mice. In contrast, the increase in Iba-1 immunoreactivity was significantly reduced in MAC1-deficient cultures (Figure 3).

To investigate the possible involvement of NOX2 in the formation and maintenance of reactive microgliosis, LPS-elicited increase of Iba-1 immunoreactivity in primary neuron-glial cultures generated from both wild-type and NOX2 deficient mice were compared. The results showed that a persistent increase in Iba-1 immunoreactivity was reduced in NOX2-deficient cultures (Figure 3). Taken together, our findings demonstrated a critical role of the MAC1/NOX2 signaling pathway in maintaining prolonged reactive microgliosis, thereby mediating persistent neuroinflammation and subsequent progressive neurodegeneration.

### 3.4. Persistent Elevation of Brain Oxidative Stress Was Ameliorated in MAC1 KO Mice

**Figure 4:**
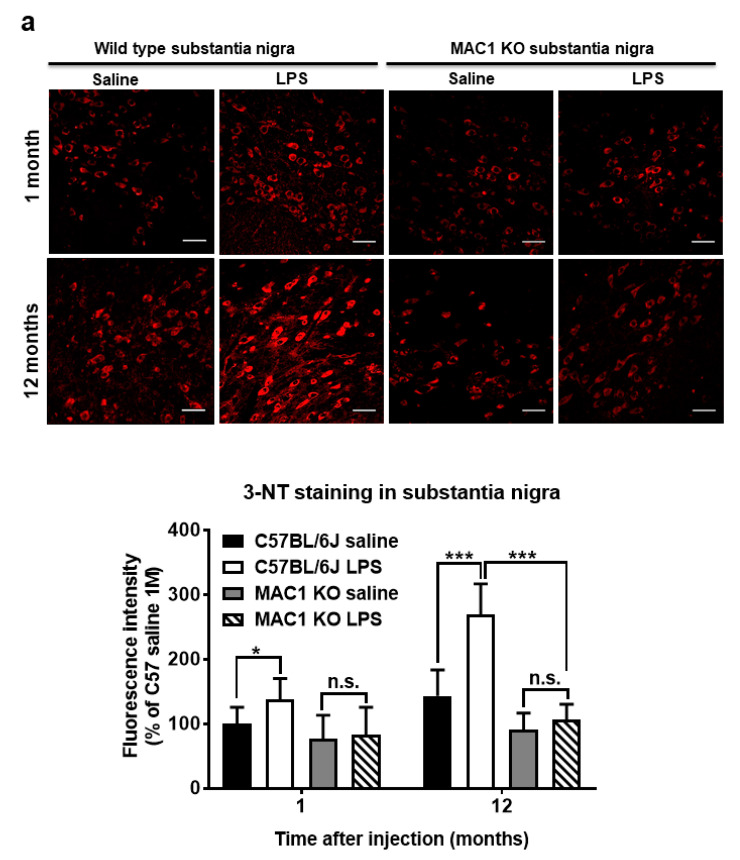

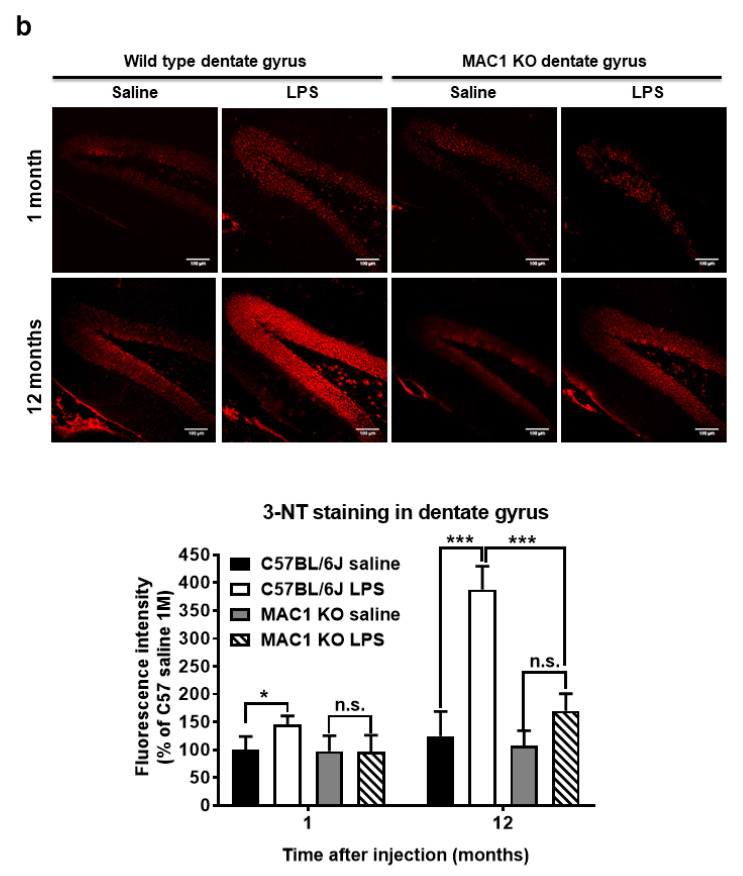
Persistent elevation of brain level of oxidative stress was ameliorated in MAC1 KO brains. Brain slides from C57BL/6J and MAC1 KO mice were collected at 1 or 12 months after saline or LPS injection (N = 3 in each group and time point). Three brain slides from each group were stained with 3-nitrotyrosine (3-NT), an oxidative stress marker. (**a**) 3-NT staining in substantia nigra and (**b**) hippocampal dentate gyrus. Scale bar in (**a**) = 50 μm, in (**b**) = 100 μm. * *p* < 0.05, *** *p* < 0.001.

To elucidate the role of MAC1 in LPS-elicited increase in oxidative stress, we compared a marker of oxidative stress 3-nitrotyrosine (3-NT), which causes nitrosylation of proteins in wild-type and MAC1 KO mouse brains. LPS caused a time-related increase in the intensity of nigral 3-NT immunoreactivity in the substantia nigra (SN) compared with saline control in wild-type mice (C57BL/6J): about 50% increase at 1 month (*p* < 0.05) and 100% at 12-month (*p* < 0.0001). However, the 3-NT level in saline- or LPS-injected MAC1 KO mouse brains did not display any difference both at 1 month or 12 months after injections (Figure 4a). In addition to the SN, we also compared changes of 3-NT immunoreactivity in other brain regions, such as the dentate gyrus of the hippocampus. Results showed the change patterns are similar to that of the SN (Figure 4b). Taken together, these data revealed that MAC1 plays an important role in sustaining microgliosis (Figure 1), producing oxidative stress, and leading to neurodegeneration (Figure 2).

### 3.5. LPS-Elicited p47^phox^ Translocation Was Reduced in MAC1 KO Microglia

**Figure 5:**
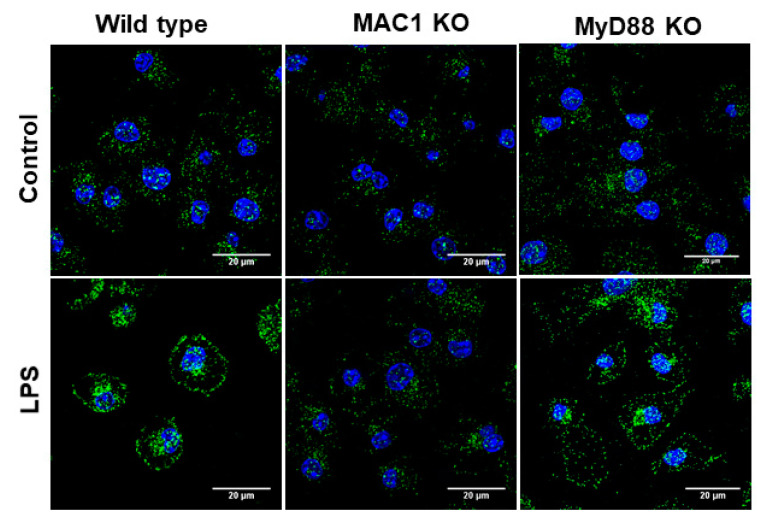
LPS-elicited p47*^phox^* translocation was reduced in MAC1 KO microglia. Microglia from wild type, MAC1 KO, and MyD88 KO were fixed and stained with p47*^phox^* 60 min after being stimulated with LPS (100 ng/mL).

Production of superoxide from microglial NOX2 requires the translocation of phosphorylated cytosolic subunits (p47*^phox^*, p67*^phox^*, and p40*^phox^*) to the cell membrane to form the active enzyme complex by binding to the membrane subunits gp91*^phox^* and p22*^phox^* [28]. To further determine whether MAC1 deficiency affects the translocation of the cytosolic subunit, we examined the membrane translocation of the cytosolic subunit p47*^phox^* by determining the level of p47*^phox^* in cytosol and membrane after stimulation with LPS in cultured microglia. In this study, we included microglia-deficient in MyD88, which is a major downstream effector of TLR-4 receptor for comparing the role of MAC1 and TLR-4 in LPS-elicited NOX2 activation. Staining results showed that LPS significantly increased the immunofluorescence intensity in the membrane of both wild-type and MyD88-deficient microglia but not in MAC1-deficient microglia (Figure 5). These results demonstrated that the activation of NOX2 is mainly mediated by MAC1 but not by TLR4 activation.

### 3.6. Prolonged Increase ERK1/2 Phosphorylation Is Associated with MAC1-NOX2 Elicited Reactive Microgliosis

**Figure 6:**
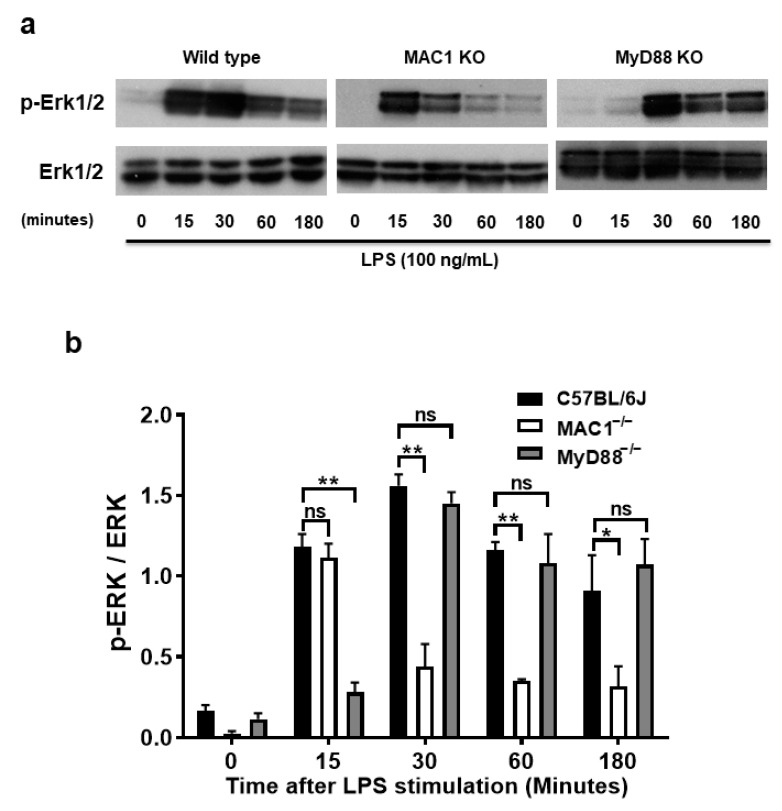

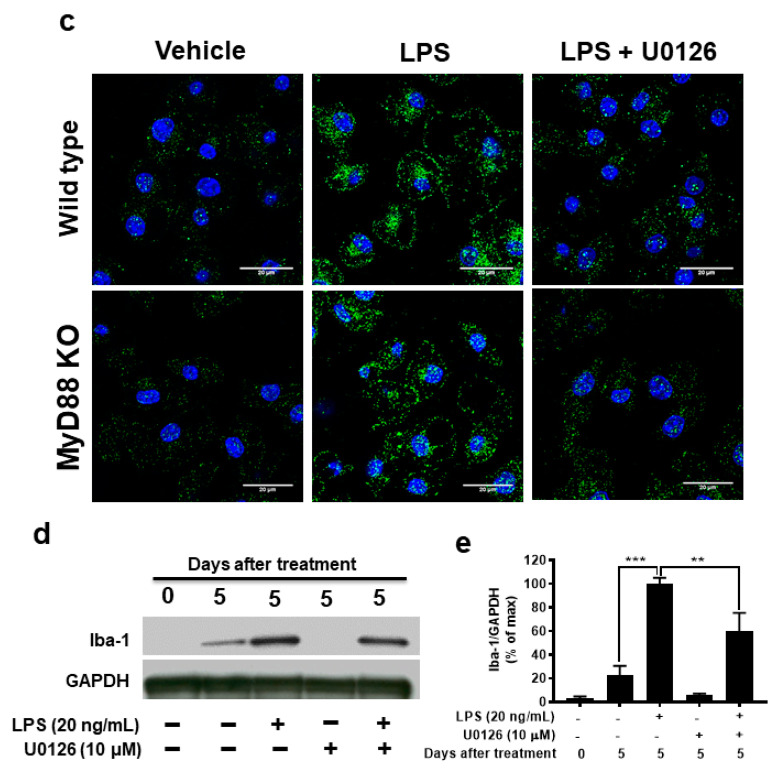
A prolonged increase in ERK1/2 phosphorylation is associated with MAC1-NOX2-elicited reactive microgliosis. Microglia from wild type, MAC1 KO, and MyD88 KO mice were treated with LPS. Cell pellets were collected at various time points for detecting the quantity of both phosphorylated-ERK1/2 or total ERK1/2 (**a**). (**b**) Quantification of ERK1/2 profiles. (**c**) Microglia from wild-type and MyD88 KO mice were stimulated with LPS for 30 min followed by Erk1/2 inhibitor U0126 (10 μM) for another 30 min. At the end of incubation, cells were fixed and stained with p47*^phox^*, scale bar = 20 μm. (**d**) Neuron-glial cultures from wild-type mice were stimulated with LPS and followed by treating with ERK1/2 inhibitor one day after LPS stimulation. Five days after U0126 treatment, the cell pellets were collected for Iba-1 Western blot analysis. (**e**) Quantification of Iba-1 density in (**d**). Data show mean ± SEM from 3 independent experiments. * *p* < 0.05; ** *p* < 0.01; *** *p* < 0.001.

The molecular mechanism underlying the coupling of MAC1-mediated NOX2 remains unclear. The purpose of the study was to search for potential signal molecules mediating p47*^phox^* phosphorylation and translocation. Among the different protein kinases screened, we found that ERK1/2 played a crucial role. We demonstrated that ERK1/2 phosphorylation in wild-type microglia was significantly increased at 15 min after LPS stimulation, peaked at 30 min, and maintained at high levels up to 3 h. By contrast, LPS-elicited ERK1/2 phosphorylation was only transiently increased at 15 min; intensities of phosphorylation rapidly declined after 30 min of stimulation. Since TLR-4 is also known involved in ERK1/2 phosphorylation, we compared the pattern of phosphorylation of MAC1- and MYD88-deficient microglia side by side. In contrast to the pattern shown in MAC1-deficient microglia, in MyD88 deficient microglia, LPS-elicited Erk1/2 phosphorylation was not observed until 30 min after LPS stimulation but was able to maintain significant high levels up to 3 h (Figure 6a,b). These data suggest that LPS-elicited increase in ERK1/2 phosphorylation during the first 15 min could be initiated by TLR4 activation, and the long-lasting of ERK1/2 phosphorylation could be mediated by MAC1 activation.

To further determine whether the long-lasting of ERK1/2 phosphorylation affects the downstream effector NOX2 activation, we stimulated both wild-type and MyD88 KO microglia with LPS for 30 min followed by treatment with ERK1/2 inhibitor U0126 (10 μM) for another 30 min. In Figure 6c, analysis of immunostaining of p47*^phox^* showed that LPS stimulation significantly increased the p47*^phox^* immunofluorescence intensity in the cell membrane. The post-treatment of ERK1/2 inhibitor, U0126, abolished the translocation of p47*^phox^* to the membrane (Figure 6c). Together, these results indicate that MAC1-elicited long-lasting phosphorylation of ERK1/2 is critical in mediating the translocation of NOX2 cytosolic subunits and its activation.

To further examine the role of ERK1/2 in LPS-induced reactive microgliosis, U0126 (10 μM) to neuron-glial cultures one day after LPS stimulation. Western blot analysis was performed 5 days after U0126 treatment. The results showed that microglial activation marker Iba-1 was significantly decreased in U0126-treated cultures (Figure 6d,e). Together, these data demonstrated that the long-lasting enhancement of ERK1/2 phosphorylation is critical in coupling the activation of MAC1 to activate microglial NOX2.

### 3.7. Inhibition of Activation of ERK1/2 Protects Dopaminergic Neurons from LPS-Elicited Toxicity

**Figure 7:**
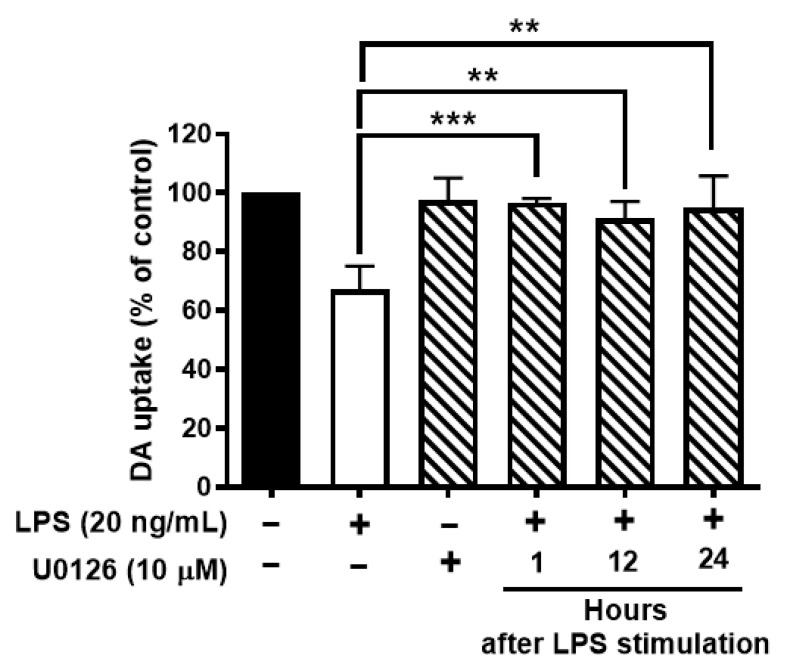
Inhibition of activation of ERK1/2 protects dopaminergic neurons from LPS-elicited toxicity. Wild-type neuron glial cultures were stimulated with LPS and ERK1/2 inhibitors were treated at 1, 12, or 24 h after LPS stimulation. On day 7 after LPS stimulation, cultures were subjected to DA uptake assay to detect the viability of DAergic neurons. Data show mean ± SEM from 3 independent experiments with 3 replicates per experiment. ** *p* < 0.01, *** *p* < 0.001.

To further test whether ERK1/2 inhibition protects neurons against LPS-mediated neuroinflammation and neurodegeneration. Neuron-glial cultures were stimulated with LPS and followed by post-treatment of U0126 at 1, 12, or 24 h after LPS stimulation. The results showed that ERK1/2 inhibition could protect DAergic neurons against LPS-induced neurotoxicity even at 24 h after LPS stimulation (Figure 7).

## 4. Discussion

**Figure 8:**
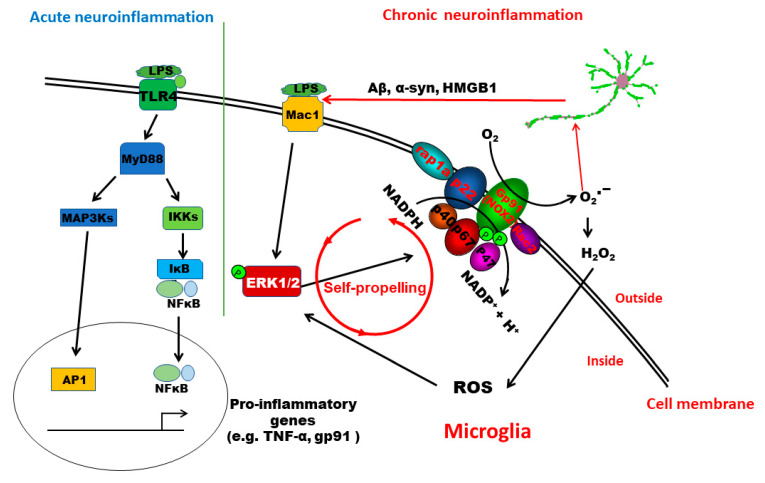
Schematic drawing depicting possible signaling pathways mediating LPS-elicited acute and chronic neuroinflammation. The TLR4 signaling pathway plays a critical role in initiating LPS-elicited acute neuroinflammation but may not be sufficient to maintain chronic neuroinflammation. This study revealed that the activation of the MAC1-NOX2-ERK1/2 pathway is essential in the initiation/maintenance of chronic neuroinflammation and in triggering the resultant neuronal loss. Further evidence indicates that the coupling of MAC1 and its downstream effector NOX2 is essential in maintaining sustained reactive microgliosis and chronic neuroinflammation. During the chronic neuroinflammatory stage, continuing activation of MAC1 by DAMPs primarily released from damaged/dying neurons triggers long-lasting ERK1/2 phosphorylation, which further activates NOX2. The MAC1-NOX2 axis activation in microglia produces superoxide and ROS to cause oxidative stress, forming a vicious feedforward cycle to maintain the reactive microgliosis. Continuing operation of this vicious cycle serves as a critical driving force to sustain low-grade neuroinflammation and drive neurodegeneration.

This study provides significant insights into understanding the signaling pathway mediating the pathogenesis of low-grade chronic neuroinflammation. We demonstrated that distinct pathways are involved in acute and chronic neuroinflammation genesis. TLR4 activation is essential for LPS-induced acute inflammation. In this paper, we have provided the first evidence indicating that MAC1 activation is needed for the transition from acute to chronic inflammation. MAC1-NOX2-ERK1/2 pathway plays a distinct role in maintaining chronic neuroinflammation and the resultant neuronal loss. Mechanistic studies revealed that the coupling of MAC1 and its downstream effector NOX2 is essential in maintaining sustained reactive microgliosis. During the chronic neuroinflammatory stage, continuing activation of MAC1 by DAMPs largely released from damaged/dying neurons triggers long-lasting ERK1/2 phosphorylation, which in turn further activates NOX2. Our evidence suggests that as a result of the activation of the MAC1-NOX2-ERK1/2 pathway, a vicious feedforward cycle is formed within reactive microglia. Continuing operation of this vicious cycle serves as a key driving force to maintain low-grade neuroinflammation and drive neurodegeneration (Figure 8).

### 4.1. TLR-4 vs. MAC1

Most of the reports studying relationships between neuroinflammation and neurodegenerative diseases have focused on TLRs-related signaling pathways because of their well-established role in launching acute inflammation. Upregulation of TLRs expression was observed in a variety of chronic neurodegenerative diseases, such as Alzheimer’s disease (TLR2 and TLR4) [29,30], multiple sclerosis (TLR 1-8) [31], and Parkinson’s disease (TLR2 and TLR5) [13,32]. However, functional roles of TLRs in these neurodegenerative diseases, in the development of chronic neuroinflammation, and in progression of neurodegeneration are still unclear. Among various TLRs, the LPS-binding TLR4 receptor has been most extensively investigated [5]. We hypothesized that TLR-4 is necessary for the initiation of acute neuroinflammation but may not be sufficient for the maintenance of chronic neuroinflammation. This study provides strong evidence supporting this possibility. LPS administered peripherally induces a strong acute inflammation in the brain, followed by the establishment of chronic low-grade neuroinflammation [12,22]. Acute inflammation requires the activation of TLR4, since the deficiency MyD88, one of the major adaptor molecule in TLR4-mediated signaling pathway, failed to mediate LPS-induced microglia activation and proinflammatory factors expression. On the other hand, we demonstrated that the initiation of acute neuroinflammation is not impaired in MAC1-deficient mice, but these mice failed to develop chronic neuroinflammation and subsequent neurodegeneration (Figure 1 and Figure 2). Taken together, our study demonstrates distinct pathways in mediating the acute and chronic neuroinflammatory process: in the LPS mouse model, TLR4 is necessary for the initiation acute neuroinflammation, while MAC1 is essential for the maintenance chronic neuroinflammation and neurodegeneration.

### 4.2. Role of the MAC-1-NOX2-ERK1/2 Signaling Pathway in LPS-Elicited Chronic Neuroinflammation

Recent studies have indicated that NOX2 is one of the key downstream effectors of MAC1 signaling in microglia [15,23,24,33]. Upon stimulation, cytosolic subunits of NOX2 translocate and bind to the cell membrane subunits to assemble the catalytically active form that produces extracellular superoxide. Here, we found that cytosolic subunit p47*^phox^* failed to translocate to the membrane in MAC1-deficient microglia but not in that of in MyD88 KO microglia after LPS stimulation (Figure 5). These results indicate that MAC1, not TLR-4, is the major receptor signaling the activation of microglial NOX2. An increment in extracellular superoxide from NOX-2 causes a gradual increase in intracellular ROS levels within neighboring cells, including neurons, through membrane-permeable metabolites, hydrogen peroxide, or peroxynitrite [2,34]. Enhanced neuronal ROS eventually drives the increase of neuronal oxidative stress at 12 months after LPS injection in wild-type but not MAC1 KO mouse brains (Figure 4). Furthermore, previous studies have demonstrated that besides LPS, MAC1 also plays a pivotal role in the chronic neuroinflammation induced by neurotoxicants, like MPTP [35], dsRNA poly I:C [36], or administration of the exogenous DAMP peptides, such as α-synuclein [26] and β-amyloid [27]. Together, this study provides strong evidence indicating a critical role of the Mac1/NOX2 signaling pathway in maintaining reactive microgliosis and elevating oxidative stress in the brain during chronic neuroinflammation.

Emerging evidence reveals that the increased intracellular ROS production leads to the activation of mitogen-activated protein kinases (MAPKs), such as Erks, c-Jun N-terminal Kinases (JNKs), or p38 MAPKs [37]. Among these MAPKs, we found that Erk1/2 is closely associated with MAC1-linked NOX2 activation but not JNKs and p38 MAPKs. The phosphorylation of JNK and p38 does not change in MAC1 KO microglia after stimulation with LPS. Activated ERK1/2 increases phosphorylation of p47*^phox^* and initiates the translocation of the cytosolic subunits of NOX2 to the membrane [38]. Here, we found that prolonged activation of Erk1/2 occurred in wild-type and MyD88 knockout microglia but not in MAC1 knockout microglia after stimulation with LPS. Consistent with the results from genetic inhibition studies, we found that the Erk1/2 inhibitor abolished the translocation of p47*^phox^*, decreased the reactive microgliosis, and displayed great potency in protecting neuronal damage against neuroinflammation-mediated neurotoxicity (Figure 6 and Figure 7). Together, our findings suggest that MAC1-NOX2 activation, ROS production, and persistent Erk1/2 activation form an intracellular self-propelling cycle to maintain the chronic reactive microgliosis (Figure 8).

From the clinical viewpoint, targeting the MAC1-NOX2-ERK1/2 axis could be an effective strategy for developing therapeutic interventions for neurodegenerative diseases. Two known anti-inflammatory compounds reduce MAC1 expression, the natural flavonoid baicalin derived from the roots and leaves of the *Scutellaria baicalensis* plant [39,40] and the synthesized leumedin NPC 15669 [41]. Unfortunately, NPC 15669 failed during Phase I clinical trials for undisclosed safety reasons. The pharmacological actions of current Erk1/2 inhibitors are not cell type-specific, which may alter the normal physiological regulation of Erk1/2 and cause unwanted effects. Thus, both MAC1 and ERK1/2 may not serve as potential therapeutic targets for neurodegenerative diseases at this stage. For this reason, our group has been investigating NOX2 as the potential target for developing therapies. So far, we have shown some promising preclinical and clinical outcomes. Inhibition of NOX2-generated superoxide could limit inflammation-driven oxidative stress and neurodegeneration [42,43,44].

## Data Availability

Data are contained within the article.
